# Larvicidal and ovicidal activities of *Artocarpus blancoi* extracts against *Aedes aegypti*

**DOI:** 10.1080/13880209.2018.1561727

**Published:** 2019-02-18

**Authors:** Maria Ruth B. Pineda-Cortel, Rachel Joy R. Cabantog, Paulo M. Caasi, Charles Anson D. Ching, Joseph Benjamin S. Perez, Paulo Gabriel M. Godisan, Cheska Marie G. Latorre, Danielle R. Lucero, Reginald B. Salonga

**Affiliations:** a Department of Medical Technology, Faculty of Pharmacy, University of Santo Tomas, Manila, Philippines;; b The Graduate School, University of Santo Tomas, Manila, Philippines;; c Faculty of Pharmacy, Meijo University, Nagoya City, Japan

**Keywords:** Dengue, mosquito, vector borne, Philippines, prevention, phytochemical

## Abstract

**Context:** Dengue control may be done by targeting its vector. In this study, we used *Artocarpus blancoi* (Elm.) Merr. (Moraceae) leaves, an endemic hematophagous insect repellent as a larvicide and ovicide.

**Objective:** We investigated the larvicidal and ovicidal activities of its soluble crude ethanol extract and the hexane, aqueous, and ethyl acetate fractions against *Aedes aegypti.*

**Materials and methods:** Third to early fourth instar *A. aegypti* larvae were exposed to 200, 400, 600, 800 and 1000 ppm of crude ethanol; to 10, 20, 40, 60 and 80 ppm of ethyl acetate; and to 500, 750, 1000, 1250 and 1500 ppm of hexane fractions of *A. blancoi*; 48 h LC_50_ and LC_90_ values were determined. For the ovicidal assay, an average of 25 eggs/paper strip was used; inhibition of egg hatchability was counted 72 h after exposure. Fractions were screened qualitatively for phytochemicals.

**Results:** Ethyl acetate soluble fraction gave the lowest LC_50_ value (18.59 ppm) followed by the crude ethanol (411 ppm), hexane (685 ppm) and aqueous (20,158 ppm) fractions. Similarly, ethyl acetate soluble fraction appeared to be the most ovicidal (80 ppm). Larvicidal and ovicidal activities of the fractions were dose dependent. Qualitative phytochemical screening revealed moderate presence of glycosides and sterols and trace amounts of triterpenes, flavonoids, saponins and tannins.

**Discussion and conclusions:***A. blancoi* is a potential larvicide and ovicide against *A. aegypti*, and future studies isolating the specific components responsible for such actions would be significant.

## Introduction

*Aedes aegypti* Linn. is a species of mosquito that requires a blood meal for egg development and is the main vector of dengue, chikungunya and yellow fever. Among these, dengue is considered one of the most prevalent vector-borne diseases in the world. The World Health Organization (WHO) estimates that 390 million infections occur yearly, with around 96 million with clinical symptoms (WHO 2018).

An important component of many disease control programs is vector control. Various pesticides and chemical formulations have been employed in an effort to control or eradicate mosquito populations. However, despite them being highly efficacious against the target species, these pesticides have the tendency to bioacccumulate in non-target organism raising concerns. Plant-derived secondary metabolites have been revealed to be interesting sources of potent insecticide.

*Artocarpus blancoi* (Elm.) Merr. (Moraceae), common name Antipolo, is widely distributed in low to medium elevations of northern Luzon to Palawan and Mindanao. This Philippine endemic plant is described as having a growth pattern size and leaf characteristics the same as that of Rimas or Breadfruit, *Artocarpus altilis* (Parkinson) Fosberg (Brown 1950). It is utilized in rope manufacture (Brown 1950), as a source of timber, and as a treatment for strangularia (Quisumbing 1978). The Aeta communities of Porac, Pampanga, Philippines, however, utilized the Antipolo tree as part of their diet and as repellent against hematophagous insects by drying and burning the plant (Ragragio et al. 2013).

Other species of the same genus have been used as insecticides. Ethanol, hexane, ethyl acetate and methanol extracts of *A. altilis* were used as insecticides against adult sweet potato weevil (Williams and Mansingh 1995); methanol extract of *A. lakoocha* Wall. Ex Roxb. (Moraceae) has proven insecticidal efficacy against second and third instar larvae of *A. aegypti.* With this previous insecticidal information of related plants, and the fact that in the Aeta communities *A. blancoi* is used as an insecticide, we have decided to pursue the study on using the plant against *A. aegypti.*

In this study, we determined the larvicidal and ovicidal activities of the soluble crude ethanol extract and the hexane, ethyl acetate and aqueous fractions obtained from the leaves of *A. blancoi.* Once proven effective, the fractions may be used in the community to control larval growth and egg hatchability, thereby controlling dengue transmission.

## Materials and methods

### Test mosquitoes

The *A. aegypti* larvae and ova used in the assay were from laboratory colony, bred at room conditions of 25–28 °C in the insectary at the Standards and Testing Division of the Department of Science and Technology Industrial Technology Development Institute (STD-DOST-ITDI), Philippines. Homogeneous populations of third and early fourth instar larvae for use in the test were obtained using disposable Pasteur pipette. Freshly laid eggs on filter paper were used in the ovicidal assay.

### Plant materials

The leaves of *A. blancoi* were obtained from Meycauayan, Bulacan, Philippines on 13 September 2015. The plant material was identified and authenticated in the University of Santo Tomas (UST) Herbarium by Ophelia S. Laurente, curator of the herbarium. Certificate of identification and authentication was dated 21 September 2015. There was no voucher specimen preserved.

### Preparation of extracts

The leaves were wiped clean, air-dried and ground to a powder using a Thomas Wiley Mill grinder. A total of 989 g pulverized *A. blancoi* leaves were percolated using analytic grade 95% ethanol for 48 h. The extracts were concentrated in a rotary evaporator then subjected to spontaneous evaporation in a water bath to obtain the crude ethanol extract. A total of 81.10 g of crude ethanol extract was obtained giving a yield of 8.2%. Liquid–liquid partitioning was employed to a portion of the crude ethanol extract using hexane and methanol–water (10; 9:1 v/v). Hexane and methanol water fractions were obtained using a separatory funnel. Both were subjected to rotary evaporation and were stored at –20 °C along with the crude ethanol extract until further testing. Further partitioning was done to the polar fraction using ethyl acetate and methanol–water (10; 1:9 v/v). Aqueous and ethyl acetate fractions were separated and were concentrated through rotary and spontaneous evaporation. Only hexane, ethyl acetate and aqueous fractions were used together with the remaining crude ethanol extract.

### Preparation of stock solutions and dilutions

Stock solution of the crude extract of 10% concentration (100,000 parts per million) was prepared by adding 100 mL of ethanol to 10 g of the crude extract. From the stock solution, subsequent doses were prepared by serial dilutions in dechlorinated water. The doses used in the test were determined by preliminary range-finding bioassay using a wide range of doses of the crude extract that gave values between 10% and 95% larval mortalities. For the crude ethanol extract, we used 200, 400, 600, 800 and 1000 ppm doses; for the ethyl acetate fraction, we used 10, 20, 40, 60 and 80 ppm; for the hexane fraction, we used 500, 750, 1000, 1250 and 1500 ppm. Same method of preparation was done for the hexane, ethyl acetate and aqueous fractions.

### Larvicidal bioassay

Larvicidal and ovicidal activities of *A. blancoi* soluble crude ethanol extract, ethyl acetate, hexane and aqueous fractions were evaluated against *A. aegypti* mosquitoes following the WHO standard protocol for testing mortality of mosquitoes with slight modifications (WHO 2005). We used disposable Pasteur pipettes to dispense a homogeneous population of third and early fourth instar larvae. A water depth in the test cups between 5.00 cm and 10.0 cm (average of 7.50 cm) was maintained to prevent undue larval mortality when soaked at deeper levels.

All test cups used in the assay were prepared prior to dosing. The appropriate volume of stock solution for each concentration was pipetted into the test cup containing the larvae. The amount of water to each cup was appropriated to the amount of test material to be added to have a final volume of 100 ± 2 mL. Mosquito larvae were exposed to a wide range of test doses to find out the activity range of the plant extracts. After 48 h of exposure, larval mortality was recorded. Each bioassay series involved five doses of each soluble extract/fraction and each dose was tested in three replicates. The bioassay was carried out twice at the same range of doses to different batches of mosquito larvae. New stock solutions were prepared at each bioassay. The negative controls used include 1% acetone in water, 1% ethanol in water, and water alone. For positive control, Abate 1SG (Abate 500E, emulsified liquid concentrate containing 500 g of organophosphate) mosquito larvicide was used.

### Ovicidal bioassay

Ovicidal activity was determined by measuring the inhibition of egg hatchability. The *A. aegypti* ova used in the assay were from laboratory colony, bred at room conditions of 25–28 °C in the insectary at the STD-DOST-ITDI. The freshly laid eggs on paper strips were observed under stereomicroscope to evaluate viability. About 20–30 laid viable eggs were gathered for each concentration of stock solution. Viable eggs were exposed to different doses of soluble crude ethanol extract, and soluble ethyl acetate, hexane, and aqueous fractions. Testing was replicated four times. The negative control cups containing 1% acetone in water, 1% ethanol in water, and water alone were maintained separately. The egg hatchability at 72 h post-treatment was observed and data were recorded.

### Phytochemical screening

The hexane, ethyl acetate and aqueous fractions were analysed for the presence of sterols using the Liebermann–Burchard test; triterpenes using Salkowski’s test; flavonoids using magnesium turning test; alkaloids using Mayer’s test; saponins using Froth test; glycosides using Fehling’s test; and tannins using ferric chloride test (Evans 2002). The fractions were tested at the STD-ITDI DOST.

### Data analysis

Percentage larval mortality was calculated by dividing the number of dead and moribund larvae by the total number of larvae introduced multiplied by 100. Microsoft Excel Data Analysis Toolpak and IBM SPSS Statistics for Windows (Armonk, NY) were used for data analysis. Pearson’s goodness-of-fit test was used to determine if the Probit model adequately fits the data provided by the experiments. The LC_50_ and LC_90_ and corresponding 95% confidence limit were determined from a log dosage Probit mortality regression line. The percentage eggs hatchability at each dose was calculated by dividing the number of hatched eggs by the total number of eggs introduced multiplied by 100. The mean hatchability at each dose was calculated including the standard deviations. One-way ANOVA was used to determine if there is significant difference in the mean hatchability among the different doses.

## Results

### Phytochemical screening

Table 1 shows the qualitative phytochemical screening results of the fractions of *A. blancoi.*

**Table 1.** Phytochemicals present in *A. blancoi* fractions.

Phytochemicals	Ethyl acetate fraction	Hexane fraction	Aqueous fraction
Sterols	(++)	(+++)	(+)
Triterpenes	(+)	(–)	(+)
Flavonoids	(+)	(–)	(+++)
Alkaloids	(–)	(–)	(++)
Saponins	(+)	(+)	(+++)
Glycosides	(++)	(+)	(+++)
Tannins	(+)	(+)	(+)

(+) presence of trace amounts of constituents, (++) moderate presence of constituents, (+++) abundant presence of constituents, (–) absence of constituents.

The results for the larvicidal and ovicidal activity of the soluble crude ethanol extract, ethyl acetate fraction and hexane fraction are summarized in Table 2. We found that the most effective larvicide and ovicide against *A. aegypti* is the soluble ethyl acetate fraction.

**Table 2.** Larvicidal and ovicidal activities of the crude ethanol extract, ethyl acetate fraction and hexane fraction of *A. blancoi*.

Dose, ppm	Mean percentage responses	
	% Larval mortality after 48 h	Eggs hatchability after 72 h
*Crude ethanol extract*		
200	17.50 ± 4.18	67.50 ± 6.61*
400	43.33 ± 9.83	45.50 ± 3.70*
600	67.50 ± 2.74	28.00 ± 4.24*
800	85.83 ± 4.92	5.50 ± 4.20*
1000	93.33 ± 6.06	0*
*Ethyl acetate fraction*		
10	20.83 ± 7.36	83.00 ± 6.16*
20	65.00 ± 5.48	73.75 ± 9.54*
40	72.50 ± 6.89	49.75 ± 8.26*
60	85.00 ± 7.07	34.25 ± 2.50*
80	93.75 ± 4.79	31.50 ± 9.04*
*Hexane fraction*		
500	31.67 ± 4.08	49.33 ± 1.15*
750	47.50 ± 4.18	51.33 ± 12.1*
1000	80.83 ± 5.85	51.67 ± 9.07*
1250	86.67 ± 5.16	39.67 ± 7.23*
1500	94.17 ± 3.76	18.00 ± 9.85*
Negativecontrol	0	96.56 ± 2.61*

Data are presented as mean ± SD.

*Significant difference on egg hatchability at different concentrations (*p* < 0.05).

The larval mortality of the soluble ethyl acetate fraction ranged from 20.83 ± 7.36 to 93.75 ± 4.79% at doses ranging from 10 to 80 ppm. The observed data do not significantly differ with the Probit model, *p* = 0.530 (Supplementary data). From this range, the LC_50_ and LC_90_ values with corresponding 95% confidence limit were estimated at 18.59 (16.10–21.02) ppm and 65.047 (55.529–79.59) ppm, respectively. No larval mortality was observed in the negative control containing 1% acetone in water. The ovicidal activity of the extract showed a decrease in egg hatchability from 83.00 ± 6.16 to 31.50 ± 9.04% at doses ranging from 10 to 80 ppm. We found that there is a significant difference in egg hatchability at different doses at a significance level of *p* < 0.05. Hatchability in the negative control was observed at 98.75 ± 2.50%.

Larvicidal activity of the soluble crude ethanol extract was observed at doses ranging from 200 to 1000 ppm, and the larvicidal mortality ranged from 17.50 ± 4.18 to 93.33 ± 6.06%. The observed data do not significantly differ with the Probit model, *p* = 0.960 (Supplementary data). From this range of larval mortality, the LC_50_ and LC_90_ with corresponding 95% confidence limit were estimated at 411 (376–445) ppm and 970 (866–1119) ppm, respectively. The negative control exhibited no larval mortality. The ovicidal activity of the soluble extract showed a decrease in egg hatchability from 67.50 ± 6.61 to 5.50 ± 4.20% at doses ranging from 200 to 800 ppm. One-way ANOVA showed that there is a significant difference in egg hatchability in different doses at a significance level of *p* < 0.05. No hatchability was observed at 1000 ppm. Hatchability in the negative control was observed at 97.25 ± 3.40%. The positive control using Abate 1SG mosquito larvicide showed LC_50_ and LC_90_ values with corresponding 95% confidence limit at 0.65 (0.59–0.71) and 1.29 (1.14–1.55), respectively.

While the larval mortality of the soluble hexane extract ranged from 31.67 ± 4.08 to 94.17 ± 3.76% at doses ranging from 500 to 1500 ppm, respectively. The observed data do not significantly differ with the Probit model, *p* = 0.979 (Supplementary data). From this range, the resulting LC_50_ and LC_90_ values with corresponding 95% confidence limit were estimated at 685 ppm (633–731) and 1343 (1232–1501) ppm, respectively. No mortality was observed in the negative control containing 1% acetone in water. The ovicidal activity of the extract showed a decrease in egg hatchability from 49.33 ± 1.15 to 18.00 ± 9.85% at doses ranging from 500 to 1500 ppm. We found that there is a significant difference in egg hatchability at different doses at a significance level of *p* < 0.05. Hatchability in the negative control was observed at 93.67 ± 2.08%.

The larval mortality of the aqueous extracts (Table 3) ranged from 15.00 ± 6.32 to 88.33 ± 6.6% at doses ranging from 10,000 to 50,000 ppm, respectively. The observed data do not significantly differ with the Probit model, *p* = 0.779 (Supplementary data). From this range, the resulting LC_50_ and LC_90_ values with corresponding 95% confidence limit were estimated at 20,158 (18,444–21,995) ppm and 55,781 (47,273–69,867) ppm, respectively. No mortality was observed in the negative control containing water alone. The ovicidal assay for the aqueous fraction was not performed due to the shortage of extract and time limitations. LC_50_ and LC_90_ values of the four extracts/fractions against *A. aegypti* larvae are summarized in Table 4.

**Table 3.** Larvicidal activity of the aqueous fraction of *A. blancoi*.

Dose, ppm	% Larval mortality after 48 h
50,000	88.33 ± 6.60
30,000	63.33 ± 9.83
20,000	53.33 ± 9.83
15,000	40.83 ± 7.36
10,000	15.00 ± 6.32
Control	0

Data are presented as mean ± SD.

**Table 4.** LC_50_ and LC_90_ values of the four extracts against *A. aegypti* larvae.

Extracts/Fractions	LC_50_, ppm (95% confidence limit)	LC_90_, ppm (95% confidence limit)
Crude ethanol	411 (376–445)	970 (866–1119)
Ethyl acetate	18.59^a^ (16.10–21.02)	65.047^a^ (55.529–79.59)
Hexane	685 (633–731)	1343 (1232–1501)
Aqueous	20,158 (18,444–21,995)	55,781 (47,273–69,867)
Positivecontrol	0.65 (0.59–0.71)	1.29 (1.14–1.55)

^a^Extract with highest efficacy as shown by low LC_50_ and LC_90_ values.

## Discussion

Botanical insecticides have been proven to be useful for the control of mosquitoes. Vector control using natural product pesticides is highly preferred than conventional pesticides because of their rapid environmental degradation and low-toxicity to other organisms (Arnason et al. 2017). The traditional sources of natural pesticides are plants since they have undergone evolutions and adaptations to improve their survival and reproduction against predators (Mann and Kaufman 2012). For this study, the extracts and fractions of *A. blancoi* leaves showed significant dose-dependent larvicidal and ovicidal activities against *A. aegypti* mosquitoes, with the ethyl acetate fraction having the highest and the aqueous fraction having the lowest efficacy.

The effectiveness of the phytochemical constituents of plants has been well documented for their larvicidal and ovicidal activity (Silva et al. 2004; Chapagain et al. 2008; Ghosh et al. 2012; Kumar et al. 2012; Arnason et al. 2017). Based on studies, all the plant constituents present in the ethyl acetate fraction of *A. blancoi* exhibit mosquitocidal properties. Sterols are proven to be toxic to some of the mosquito species thus its possible utilization in vector control (Ghosh et al. 2012). Terpene compounds exhibit larvicidal activity as well by the blockage of the sterol carrying protein (Kumar et al. 2012). Saponins act as a pest control agent because of their capability to cause increased mortality levels, lowered food intake, weight reduction, retardation, disturbances in the development, and decreased reproduction in pests. They make the food less attractive to eat, block sterol uptake by forming insoluble complexes, induce digestive problems, and cause molting defects to insects (Silva et al. 2012). Mosquitocidal activity has also been shown by saponins as demonstrated by the study by Chapagain et al. (2008) wherein saponins from a root-derived callus of *Balanites aegyptiaca* (L.) Delile (Zygophyllaceae) showed larvicidal activity against *A. aegypti* larvae. Glycosides, particularly cyanogenic glycosides, exhibit insecticidal properties through the production of hydrogen cyanide when metabolized which causes the inhibition of cytochrome oxidase and other respiratory enzymes (Yu 2015). Larvicidal, ovicidal and repellent activities are present in extracts of *Calotropis gigantean* (L.) W. T. Alton (Apocynaceae), which contain glycosides as one of the major phytochemical groups (Kumar et al. 2012). Tannins from the extract isolated from *Magonia pubescens* (Merrill) Figlar & Noot. (Sapindaceae) also showed larvicidal activity against *A. aegypti* larvae (Silva et al. 2004). These phytochemicals are responsible for the insecticidal efficacy of the ethyl acetate fraction of *A. blancoi* against *A. aegypti* larva and ova.

Studies have also been made regarding the insecticidal activity of other *Artocarpus* species. Glover (2015) studied the insecticidal and repellent activities of *A. altilis* male inflorescence against *A. aegpyti* and revealed that the methanol extract showed significantly greater toxicity to mosquitoes (Glover 2015). In addition, Williams and Mansingh (1995) found that *A. altilis* leaves exhibited insecticidal activity on *Cylas formicarius*, a sweet potato weevil. The results showed that the active compounds were found on the nonpolar extract of the leaves, which was a pentacyclic triterpene with unsaturation at the 12–13 position. In this study of Williams and Mansingh (1995), the ethyl acetate fraction was inactive, which is contrary with the results of the present study on *A. blancoi*. Upadhyay (2013) conducted a study regarding *Artocarpus heterophyllus* (Lamarck) and showed that the hexane, methanol, petroleum ether and chloroform extracts have caused very high larval mortality and significant reduction in larval weight in comparison with the control. The mentioned studies have proven the insecticidal activity of other *Artocarpus* species.

Variations in the insecticidal activities of plant extracts may be due to several factors. For instance, the efficacy of phytochemicals against insects can vary significantly depending on plant species, plant parts used, age of plant parts (young, mature or senescent), and the target vector species. One study focused on the current state of knowledge on phytochemical sources and mosquitocidal activity with reference to the mechanism of action on target population, variation due to species of mosquito, instar specificity, solvent polarity, and the nature of active ingredient present in the plant. Moreover, the study demonstrated that extraction of active biochemical compounds from plants depends on the polarity of solvents used. Different solvent types can significantly affect the potency of extracted plant compounds, and moderately polar solvents like ethyl acetate were also reported to provide good bioassay results (Ghosh et al. 2012).

The low bioactivity of the aqueous fraction may be attributed to various factors. The aqueous extract exhibited abundant amounts of flavonoids, saponins and glycosides, moderate amounts of alkaloids, and trace amount of sterols, triterpenes and tannins. This fraction contained more phytochemical constituents than the hexane and ethyl acetate fractions. Adwan et al. (2011) described the antagonistic effect of compounds in plant extracts. The study showed that as the number of compounds in the extract increases, there is a possibility of one compound interfering with the action of another compound in the extract. Another study showed the importance of sequentially extracting crude samples to minimize the number of compounds present in the extract. The study reiterated that the more compounds present in the extract are, the more antagonistic activities may be observed (Uthayarasa et al. 2010). Furthermore, the varying polarities of the plant constituents may have also contributed to the low activity of the aforementioned. This may be due to the possibility that the most bioactive compound which demonstrated the highest larvicidal and ovicidal properties are semipolar in nature, thereby rendering the ethyl acetate fraction most effective.

## Conclusions

Based on the findings of the study, the researchers hereby make the following conclusions: (1) *A. blancoi* extract has larvicidal and ovicidal activities against *A. aegypti*; (2) among the fractions, the soluble ethyl acetate fraction exhibited greatest toxicity to both *A. aegypti* larvae and ova; (3) the phytochemical screening of the fractions revealed the presence of aromatic secondary metabolites which include sterols, saponins, glycosides, tannins, flavonoids and alkaloids. Further studies on the application of *A. blancoi* extracts may be done to assess its effectiveness in the community.
